# Spontaneous PICC migration after cardiac surgery: a case report

**DOI:** 10.1093/ehjcr/ytag638

**Published:** 2026-09-10

**Authors:** John P Tsoumpelis, Samuel J Martin, Marek Polomsky

**Affiliations:** Department of Surgery, SUNY Upstate Medical University, 750 East Adams Street, NY 13210, USA; Department of Surgery, SUNY Upstate Medical University, 750 East Adams Street, NY 13210, USA; Department of Surgery, SUNY Upstate Medical University, 750 East Adams Street, NY 13210, USA

**Keywords:** Cardiac surgery, Coronary artery bypass grafting, Peripherally inserted central catheter migration, Case report, Ventricular tachycardia, Cardioversion, Postoperative cardiac surgery complication

## Abstract

**Background:**

Peripherally inserted central catheters (PICCs) are frequently used in cardiac surgery patients to provide reliable long-term venous access for vasoactive medications, parenteral nutrition, and prolonged intravenous therapies. Although PICCs are associated with fewer complications than traditional central venous catheters, spontaneous catheter migration remains an uncommon but clinically important event that may lead to catheter malfunction or vascular injury.

**Case summary:**

We report the case of a 73-year-old man with multivessel coronary artery disease who underwent coronary artery bypass grafting ×3 with left atrial appendage ligation. A PICC placed preoperatively through the right basilic vein had its tip confirmed at the cavoatrial junction. On postoperative day 7, the patient developed haemodynamically unstable ventricular tachycardia following a prolonged coughing episode and underwent synchronized cardioversion. A chest radiograph obtained 6 h after prior confirmation of appropriate positioning demonstrated upward migration of the PICC tip into the right internal jugular vein. The catheter was removed and replaced without complication.

**Discussion:**

This case highlights postoperative coughing and synchronized cardioversion as potential contributors to PICC migration in cardiac surgery patients and underscores the importance of reassessing catheter position following high-risk events to prevent complications related to catheter malposition.

Learning points

- Incidentally discovered PICC migration after cardiac surgery may represent a clinically significant near-miss event, with potential harm from continued catheter use.
- Prolonged coughing, intensive pulmonary physiotherapy, synchronized cardioversion, and prolonged catheter dwell time may have contributed to PICC migration.

## Introduction

Cardiac surgery patients may require peripherally inserted central catheters (PICCs) for vasoactive medications, total parenteral nutrition (TPN), prolonged antibiotic therapy, or poor peripheral intravenous access. Although generally safe, PICCs may be associated with complications including deep vein thrombosis, peripheral nerve injury, vessel injury, phlebitis, and bleeding.^1^ Spontaneous PICC migration is less common but clinically important because catheter malposition may remain undetected until adverse consequences occur.

Optimal PICC tip positioning is the lower third of the superior vena cava (SVC) at the cavoatrial junction, confirmed by intracavitary ECG or chest X-ray.^1–3^ Spontaneous PICC migration is unintended catheter-tip displacement from this position to another location within the venous system. The most reported sites of spontaneous PICC migration include the internal jugular, subclavian, and brachiocephalic veins.^4^ One study reported spontaneous migration in 4.1% of PICC placements in an oncologic patient population.^5^

Known contributing factors to PICC migration include increased intrathoracic pressure, deep inspiration, and excessive arm movement.^6,7^ Postoperative cardiac surgery patients are frequently encouraged to cough and perform deep breathing exercises to promote pulmonary hygiene and prevent complications such as atelectasis and pneumonia. In addition, intense skeletal muscle contractions and abrupt arm movements can occur in cardiac surgery patients when they undergo synchronized cardioversion. These routine postoperative respiratory and antiarrhythmic interventions may contribute to spontaneous PICC migration. We present a case of incidentally discovered spontaneous PICC migration into the right internal jugular vein after cardiac surgery, detected shortly after prolonged coughing and synchronized cardioversion. This case highlights the potential consequences of undetected catheter malposition and the importance of continued vigilance in high-risk postoperative patients.

## Summary figure

**Figure:**
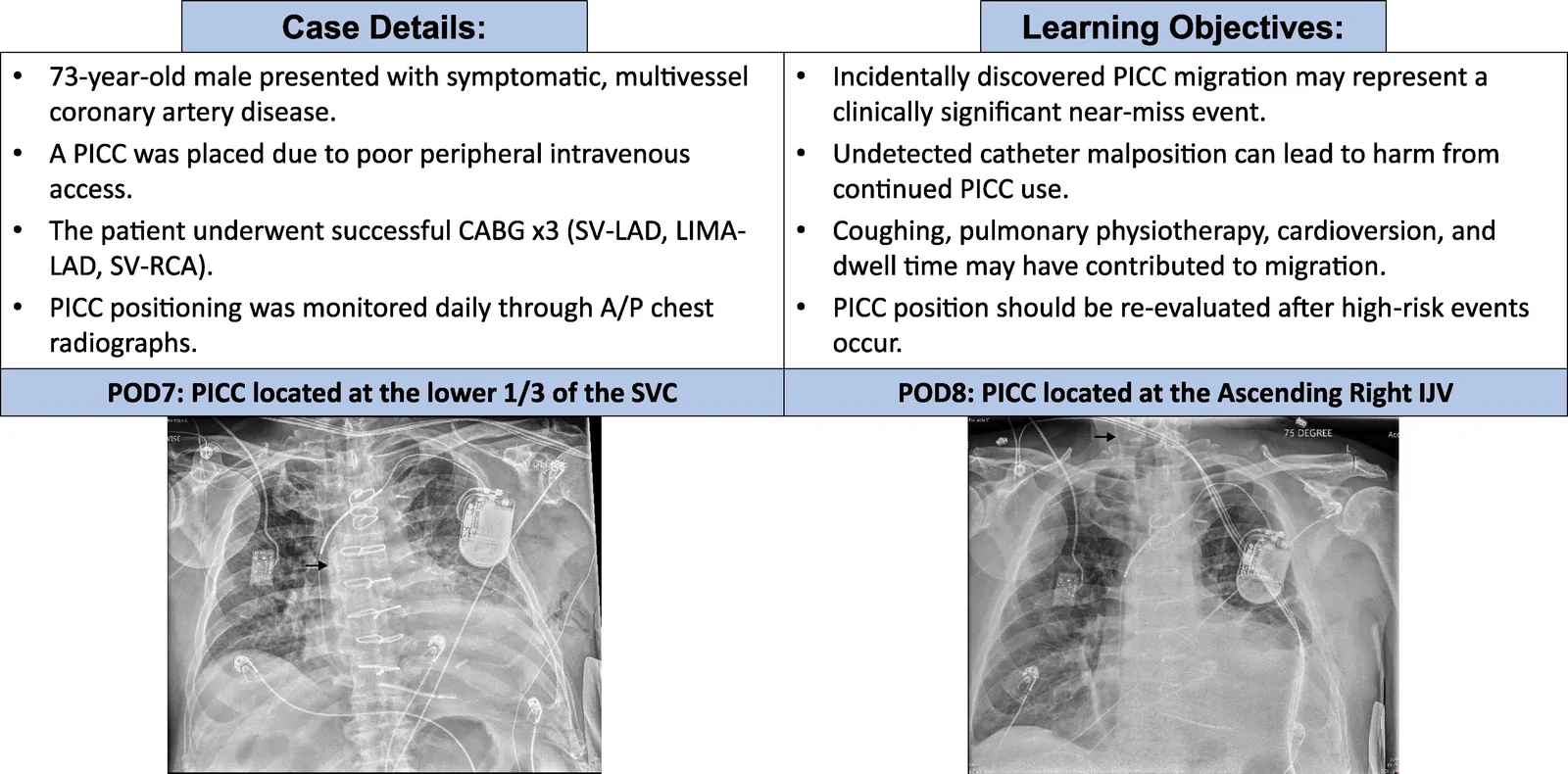


## Case presentation

A 73-year-old male with obesity, prior myocardial infarction, congestive heart failure, atrial fibrillation, type 2 diabetes, hypertension, and hyperlipidaemia presented with severe multivessel coronary artery disease. Coronary angiography revealed 70% stenosis of the proximal left anterior descending (LAD) artery, 90% stenosis of the distal LAD, and 95% stenosis of the proximal right coronary artery. The patient subsequently underwent coronary artery bypass grafting ×3 with left atrial appendage ligation via median sternotomy.

Eight days prior to surgery, a PICC was placed due to poor peripheral intravenous access. The catheter was inserted through the right basilic vein with the tip positioned at the cavoatrial junction in the lower third of the SVC. A 40-cm catheter was used, with no external length at the insertion site. Tip placement was confirmed using the 3CG tip location system (Bard Access Systems, Salt Lake City, UT, USA), with intracavitary electrocardiography (IC-ECG) demonstrating maximum P-wave amplitude without negative deflection. Chest radiography was not required for confirmation. The PICC position was monitored through daily anterior–posterior (AP) chest radiographs.

**Figure 1:**
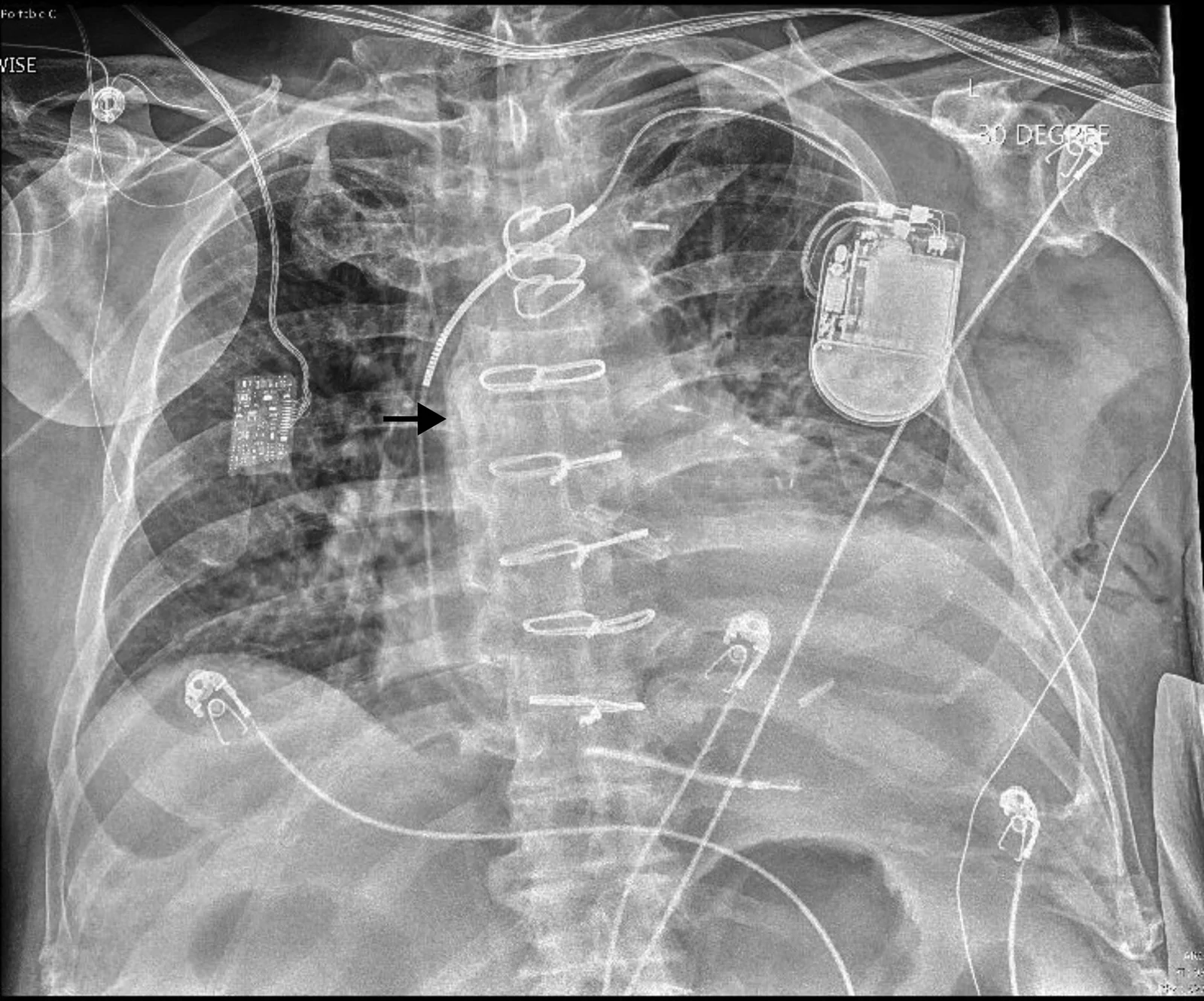
Anterior–posterior chest radiograph taken on postoperative day 7 showing the peripherally inserted central catheter tip in the lower third of the superior vena cava at the cavoatrial junction (black arrow).

On postoperative day (POD) 7, 15 days after PICC placement, the patient continued intensive pulmonary physiotherapy, including incentive spirometry, directed coughing, and deep breathing exercises. Later that evening, the patient experienced a prolonged episode of ventricular tachycardia (VT). A complete set of laboratory studies was successfully obtained through the PICC line. Chest radiography performed at that time revealed no new intrathoracic pathology and confirmed appropriate PICC positioning (*Figure 1*).

**Figure 2:**
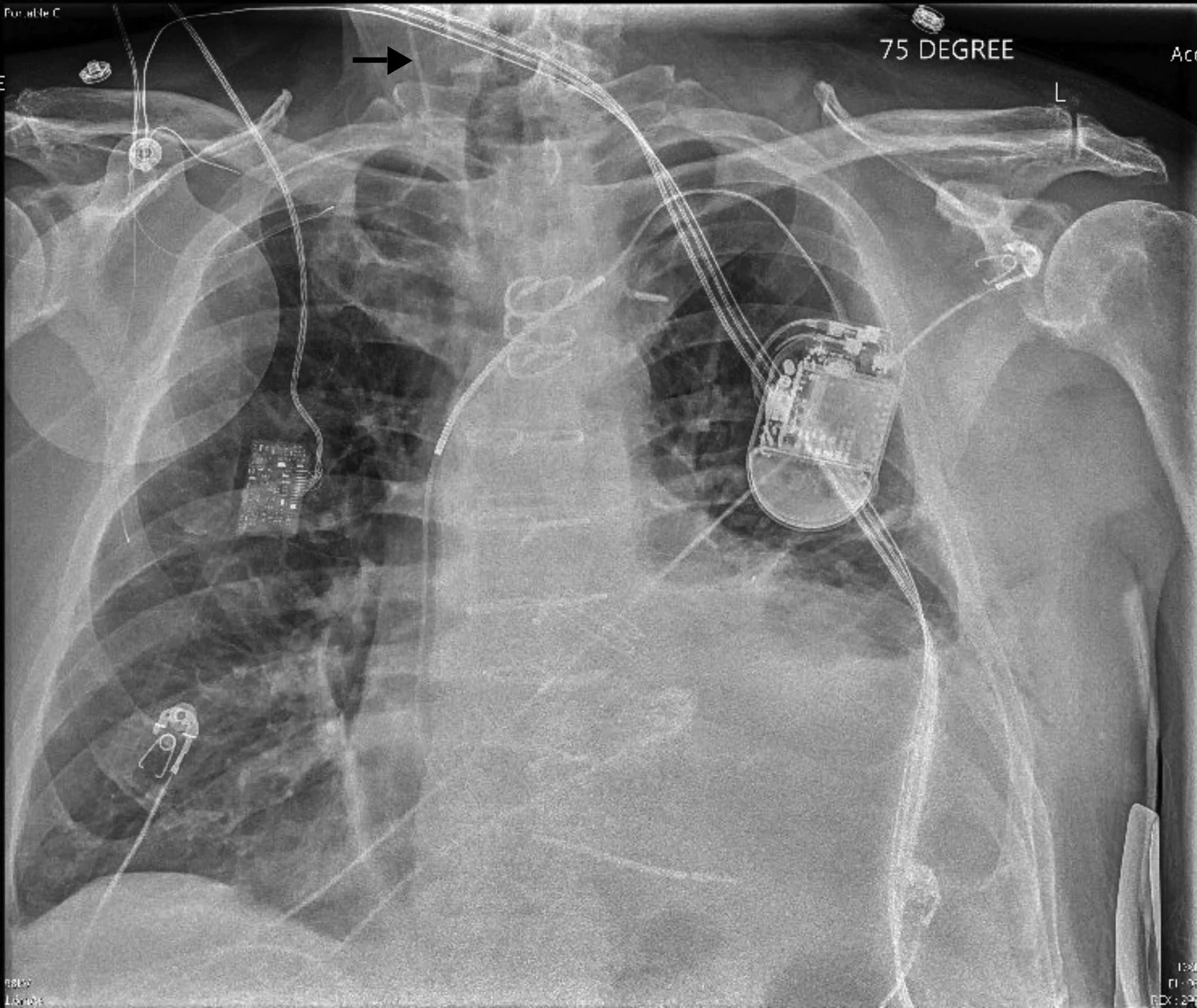
Anterior–posterior chest radiograph taken on postoperative day 8 (6 h after prior radiograph) showing peripherally inserted central catheter malposition with an upward migration into the right internal jugular vein (black arrow).

The patient then had an episode of prolonged coughing, light-headedness, and remained in VT becoming haemodynamically unstable. The patient was sedated and subsequently underwent synchronized cardioversion at 200 J, which restored sinus rhythm and haemodynamic stability. On POD 8, 6 h following the previous AP chest radiograph, a repeat chest radiograph revealed PICC malpositioning. The catheter had migrated upward into the right internal jugular vein, with clear distinction of new tip location (*Figure 2*). There was no attempt to reposition the PICC, and it was removed. A new PICC was later inserted into the right basilic vein. The remainder of the patient’s hospital course was uneventful, and he was discharged home on POD 12. The patient was seen at his 1-month postoperative visit and was recovering well from surgery.

## Discussion

In the early postoperative period, cardiac surgery patients are at increased risk of pulmonary complications including pneumonia, pleural effusion, pneumothorax, atelectasis, acute respiratory distress syndrome, and prolonged mechanical ventilation.^8^ To reduce these risks, intensive pulmonary physiotherapy, including incentive spirometry, directed coughing, and deep breathing exercises, is routinely implemented. However, coughing increases intrathoracic pressure, while deep inspiration generates marked negative intrathoracic pressure; both are recognized risk factors for spontaneous PICC migration. In addition, the PICC had been in place for 15 days when migration was identified, and prolonged catheter dwell time may have represented an additional risk factor. Together, these factors may make postoperative cardiac surgery patients vulnerable to catheter displacement, although the relative risk compared with other populations remains uncertain.

Another notable feature of this case is the close temporal association between synchronized cardioversion and subsequent identification of PICC migration. Tan *et al*. reported a 45-year-old male who sustained anterior shoulder dislocation, a Bankart lesion, and supraspinatus tear following synchronized cardioversion.^9^ These injuries occurred while under propofol-induced anaesthesia, demonstrating the significant skeletal muscle contraction and arm movement that can result from cardioversion. Excessive arm movement is a known risk factor for PICC migration. Therefore, it is plausible that involuntary muscle contraction and arm motion during synchronized cardioversion may have contributed to our patient’s spontaneous PICC dislodgement into the right internal jugular vein. To our knowledge, cardioversion-associated PICC migration has not been well described. However, causality cannot be established in this case because prolonged coughing, intensive pulmonary physiotherapy, prolonged catheter dwell time, and synchronized cardioversion occurred within the same short time window. Rather than identifying a definitive mechanism, this case raises awareness that abrupt physiologic or mechanical stressors may contribute to catheter migration in susceptible patients.

Overall, the central clinical concern in this case is that PICC migration was detected incidentally before the patient developed symptoms directly attributable to malposition. Without routine radiographic monitoring, the catheter could have remained in use despite migration into the right internal jugular vein. Continued infusion of vasoactive medications, TPN, hyperosmolar medications, or other irritant therapies through a malpositioned catheter could have increased the risk of local vasospasm, chemical thrombophlebitis, vascular injury, inadequate medication delivery, and other catheter-related complications. Early detection and removal therefore transformed a potentially harmful event into a near miss.

## Conclusion

This case highlights an incidentally discovered near-miss event involving spontaneous PICC migration into the right internal jugular vein in a postoperative cardiac surgery patient. Although causality cannot be determined, prolonged coughing, intensive pulmonary physiotherapy, synchronized cardioversion, and 15-day catheter dwell time may have contributed to migration. Since PICC malposition may not produce immediate patient symptoms, continued vigilance, and radiographic assessment after high-risk clinical events may help prevent harmful complications from infusion through an improperly positioned catheter.

## Lead author biography


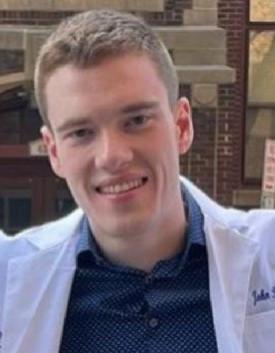


John P. Tsoumpelis is a third-year medical student at SUNY Upstate Medical University in Syracuse, New York. His research interests include cardiovascular medicine, perioperative complications in surgery, and postoperative patient management. He is involved in clinical research examining outcomes in cardiac surgery as well as pain management strategies for postoperative burn surgery patients. He intends to pursue residency training in a procedure-oriented specialty while continuing to contribute to clinical research.

## Data Availability

The data underlying this article are available within the article and its accompanying figures.
